# Indocyanine Blue (ICB) as a Functional Alternative to Indocyanine Green (ICG) for Enhanced 700 nm NIR Imaging

**DOI:** 10.3390/ijms252413547

**Published:** 2024-12-18

**Authors:** Atsushi Yamashita, Paul Jang, Kai Bao, Satoshi Kashiwagi, John V. Frangioni, Hak Soo Choi

**Affiliations:** 1 Gordon Center for Medical Imaging, Department of Radiology, Massachusetts General Hospital and Harvard Medical School, Boston, MA 02114, USA; 2 Curadel Pharma, Inc., Natick, MA 01760, USA

**Keywords:** dual-channel imaging, near-infrared dye, rapid clearance, hepatobiliary excretion

## Abstract

Despite significant advancements in bioimaging technology, only a limited number of fluorophores are currently approved for clinical applications. Indocyanine green (ICG) is the first FDA-approved near-infrared (NIR) fluorophore and has significantly advanced clinical interventions over the past three decades. However, its single-channel imaging at 800 nm emission is often insufficient for capturing comprehensive diagnostic information during surgery. In this study, we evaluate indocyanine blue (ICB), an ICG analog with a shorter polymethine bridge, as a promising candidate for multi-channel NIR imaging. ICB demonstrated peak absorption and emission approximately 100 nm shorter than ICG in aqueous solutions, placing it within the 700 nm range of the NIR window. Furthermore, ICB exhibited favorable solubility and optical properties in aqueous environments, supporting its potential for in vivo imaging applications. Notably, ICB shows rapid systemic clearance, likely due to its lower molecular weight, which facilitates clear visualization in angiography, cholangiography, and lymph node mapping with minimal background interference. Additionally, dual-channel imaging of tumors and lymph nodes was achieved using a tumor-targeting fluorophore in conjunction with ICB, illustrating the potential for enhanced intraoperative guidance. ICB emitting at 700 nm, therefore, can be useful in NIR imaging, broadening the possibilities for improved diagnostic accuracy and therapeutic outcomes in clinical settings.

## 1. Introduction

Near-infrared (NIR) fluorescence imaging has significantly advanced medical image-guided interventions over the past three decades, providing surgeons with an enhanced understanding of anatomical, physiological, and pathological contexts [1,2,3,4,5,6,7,8,9,10]. This is extremely important not only for conventional surgery but also for robotic surgery. The integration of imaging data into artificial intelligence (AI) systems for applications such as pathological diagnosis, intraoperative guidance, and autonomous surgical robotics underscores the transformative potential of these technologies in the future of healthcare [11]. Given the extensive data required to train AI systems, a diverse array of imaging data spanning multiple fluorophore wavelengths will be essential to optimize the effectiveness of image-guided surgery [12,13].

Indocyanine green (ICG), a water-soluble yet hydrophobic heptamethine cyanine dye, was the first NIR fluorophore approved for clinical use by the U.S. FDA in 1959 [14,15]. Due to the limited availability of clinically approved fluorescence imaging dyes, “fluorescence-guided surgery” has become synonymous with ICG, and until recently, nearly all available NIR cameras focused solely on the 800 nm wavelength [16,17,18,19,20,21,22,23,24,25,26,27]. Indeed, pafolacianine (Cytalux^®^) was approved by the FDA for ovarian and, subsequently, lung cancer surgeries [28,29,30,31]. This agent peaks at 775 nm and 796 nm for absorption and emission, respectively [32], leveraging the widespread use of 800 nm NIR imaging systems.

The NIR window, with an emission range between 650 nm and 1700 nm, represents an optical window that leverages NIR fluorescence for medical imaging [33,34,35,36,37,38,39]. Notably, silicon camera sensitivity declines sharply beyond 800 nm, and wavelengths above 900 nm pose additional challenges, including diminished sensitivity, limited availability of stable agents, and chemical instability [40]. Armed with this optical window, dual-channel imaging systems were developed to obtain maximal imaging information within the 650–900 nm range. For example, the FLARE imaging systems facilitate real-time, simultaneous acquisition of color video and two independent NIR channels centered at 700 nm and 800 nm [41]. By employing fluorophores with two distinct chemical structures, simultaneous yet independent visualization of two distinct biological targets is enabled. For example, we previously visualized thyroid and parathyroid glands [42], bile ducts and arteries [43], pan and sentinel lymph nodes [44], bone and cartilage [45], and cancer and tumor vasculature [46] within the same subject.

Despite the availability of some intraoperative optical imaging systems, the demand for clinically useful NIR fluorophores continues to grow. Simultaneous imaging of neighboring but distinct tissues, such as nerves, vasculature, and lymphatics, remains a clinical challenge due to anatomical variability and the complexity of identifying structures in high-risk cases [47,48]. Given that most tumor-targeted NIR fluorophores currently used in clinical settings are optimized for the 800 nm channel, the development of fluorophores targeting the 700 nm channel is critical. Since ICG has already been widely studied and applied in clinical settings, an ideal approach would involve modifying ICG to create a fluorophore optimized for the 700 nm wavelength channel. The length of the polymethine bridge has been well-known as the key to tuning the excitation/emission profile of the dye [49], and extending this bridge shifts the emission further into the NIR but typically lowers quantum yields [50,51].

In this study, we investigated indocyanine blue (ICB) as a functional alternative to ICG with a shorter polymethine bridge for enhanced 700 nm NIR imaging. A previous publication on ICB by the Smith group [52] laid the foundation for this work and introduced ICB for 700 nm NIR imaging. Our study extends this pioneering work to include full optophysicochemical characterizations of ICB both in vitro and in vivo, offering a direct quantitative comparison with ICG. We also provide a solution for improved image-guided surgery by preventing vascular injuries and ensuring the accurate identification of lymph nodes during tumor resections. ICB has the potential to serve as a valuable addition to the NIR imaging toolkit, providing significantly enhanced tumor diagnostics, facilitating AI-based surgical training, and improving the safety of surgical interventions.

## 2. Results

**Figure 1:**
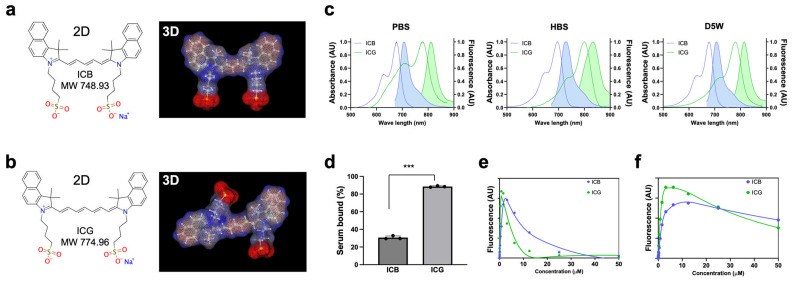
Chemical structures and optophysical properties of ICB (blue) and ICG (green). The 2D and 3D structures of ICB (**a**) and ICG (**b**) were generated by MarvinSketch (ChemAxon). (**c**) Absorbance and fluorescence spectra were measured in PBS, HBS, and D5W solutions. (**d**) Plasma protein binding assay of fluorophores compared with ICG incubated in HBS for 4 h. Data are expressed as means ± SDs (*n* = 3; *** *p* < 0.001). (**e**,**f**) ICB and ICG quenching patterns in terms of concentrations measured in the 90° geometry using a 660 nm (for ICB) or 760 nm (for ICG) NIR laser diode light source (quenching by laser) (**e**); or in the 0° geometry using the FLARE imaging system (**f**). Photobleaching curves were obtained by incubating different concentrations of dyes.

**Optophysical Properties:** The molecular weight (MW) of ICB is 748.93 Da, while ICG is 774.96 Da due to the longer polymethine chain. Interestingly, ICB showed a completely flat planar 3D backbone structure (Figure 1a), while ICG displayed a twisted, non-planar conformation (Figure 1b). Upon measurement of the optical and physicochemical properties, ICB and ICG showed absorbance and fluorescence spectra in the 700 and 800 nm ranges, respectively, in all aqueous solvents tested in this experiment, including distilled water (DW), saline, phosphate-buffered saline (PBS), 10% fetal bovine serum supplemented with 50 mM HEPES (HBS), and dextrose 5% in water (D5W) (Table 1). Due to the shorter resonance structure of delocalized electrons, pentamethine ICB displays absorption and emission maxima 100 nm shorter than those of ICG. The peak absorption and emission of ICB in DW, saline, PBS, and D5W are almost the same, while the absorption and emission peaks in HBS were red shifted around 20 nm for both ICB and ICG (Figure 1c). Additionally, the fluorescence quantum yield (QY) of both dyes in HBS is significantly higher than in other aqueous solvents, likely due to interactions with serum proteins that reduce H-aggregates in the fluorophores [52]. In all aqueous solutions, ICB displays higher QY compared to ICG. The extinction coefficients (ε) of both ICB and ICG are similar, around 200,000 M^−1^ cm^−1^, except for ICG in saline (139,500 M^−1^ cm^−1^) and PBS (138,600 M^−1^ cm^−1^). The solubility of ICB in HBS is significantly lower compared to ICG, possibly due to the weaker interactions with serum proteins (Figure 1d), which may affect solubilization (Table 1). To investigate these properties further, we conducted a quenching assay in HBS. Quenching occurred at 1.5 μM for ICB and 0.78 μM for ICG when excited at a 90° angle between excitation and detection (Figure 1e). Under the 0° geometry of the intraoperative FLARE imaging system, the peak concentrations shifted to ≈10 μM for ICB and ≈5 μM for ICG, with a slow rate of quenching as concentration increased (Figure 1f), a pattern consistent with previous findings in heptamethine zwitterionic dyes [53]. Collectively, these optical properties of ICB in aqueous solvents support its suitability for subsequent in vivo imaging assays.

**Figure 2:**
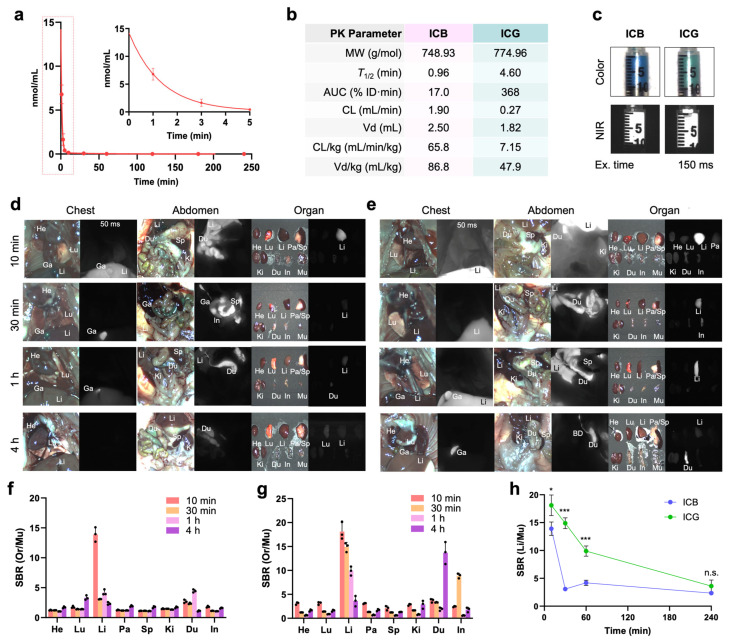
In vivo pharmacokinetics and biodistribution of ICB and ICG. (**a**) The plasma concentration curve of ICB and (**b**) pharmacokinetic parameters of ICB and ICG were calculated. (**c**) CD-1 mice were intravenously injected with 25 nmol of each sample before imaging, and the NIR fluorescence images of ICB (**d**) and ICG (**e**) were recorded intraoperatively. Abbreviations used are Bl, bladder; Du, duodenum; In, intestine; Ki, kidneys; Li, liver; Lu, lungs; Pa, pancreas; Sp, spleen. The signal-to-background ratio (SBR) of ICB (**f**) and ICG (**g**) in resected organs against muscle was measured using ImageJ analysis. (**h**) Intraoperative imaging of ICB and ICG in the liver over the period of 240 min. Data are expressed as means ± SDs (*n* = 3; * *p* < 0.05, *** *p* < 0.001; n.s. = not significant).

**In Vivo Pharmacokinetics and Biodistribution:** Since ICG is widely used in clinical imaging applications, such as angiography, cholangiography, lymphatic vessel mapping, and tumor imaging, ICB also holds significant potential for these purposes, particularly within shorter wavelength (700 nm) channels. Before advancing to in vivo imaging applications, we evaluated the basic pharmacokinetics and biodistribution of ICB in CD-1 mice up to 4 h after a single intravenous injection (Figure 2). To obtain the blood concentration decay curves for ICB, we measured NIR fluorescence signal intensities in mouse serum collected at predetermined intervals. Both ICB and ICG exhibited pharmacokinetics consistent with the single-compartment model, clearing rapidly from the bloodstream to the liver (Li) and gallbladder (Ga). However, compared to ICG, ICB displayed a shorter half-life (0.96 min), a smaller area under the curve (AUC), a larger volume of distribution (2.5 mL), and a faster clearance rate (65.84 mL/min/kg). This faster clearance is likely due to the lower molecular weight of ICB, resulting from its shorter polymethine bridge, which suggests efficient hepatobiliary clearance.

The biodistribution profiles of ICB and ICG were similar in the thoracic and abdominal cavities and across resected organs, except for a significantly higher liver signal observed with ICG (Figure 2d,e). Additionally, the signal-to-background ratio (SBR) in resected organs, including the heart, lung, liver, pancreas, spleen, kidneys, duodenum, intestine, and muscle, indicated sustained ICG fluorescence signals from the liver, even at 4 h post-injection (Figure 2g,h). In contrast, ICB exhibited lower liver signals and reduced background fluorescence, suggesting efficient biliary clearance. These imaging results are consistent with the blood half-life data, supporting ICB’s rapid clearance and lower background signal relative to ICG. Overall, ICB demonstrates a promising pharmacokinetic profile, with rapid clearance and lower background signals compared to ICG, indicating its potential as an effective fluorophore for clinical imaging applications, especially in finding liver metastases.

**Figure 3:**
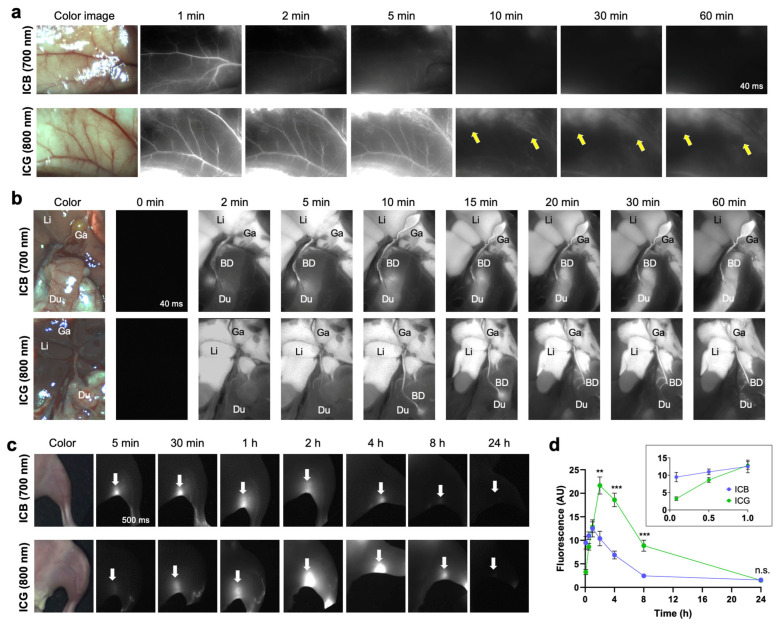
In vivo NIR fluorescence images of ICB and ICG in the vessel, gallbladder, and lymph nodes. (**a**) NIR angiography using ICB and ICG up to 5 min post-intravenous injection. Yellow arrows indicate background uptake signals. (**b**) NIR cholangiography using ICB and ICG up to 10 min post-intravenous injection. Abbreviations used are BD, bile duct; Du, duodenum; Ga, gallbladder; Li, liver. (**c**) NIR lymph node mapping of popliteal lymph nodes (white arrows) using ICB and ICG up to 24 h post-intradermal injection. (**d**) Longitudinal monitoring of signal changes of ICB and ICG in the popliteal lymph nodes from the images shown in (**c**). Data are expressed as means ± SDs (*n* = 3; ** *p* < 0.01, *** *p* < 0.001; n.s. = not significant).

**NIR Angiography, Cholangiography, and Lymph Node Mapping:** ICG has proven beneficial in clinical practice for delineating vascular, lymphatic, and hepatobiliary structures [54]. We systemically compared ICB and ICG in angiography, cholangiography, and lymph node mapping (Figure 3). Angiography is a valuable diagnostic method not only for assessing blood perfusion but also for highlighting inflammation and anatomical changes in various tumor types. Clear fluorescence signals from both ICB and ICG were observed immediately (within 1 min) following a single intravenous injection of each dye (Figure 3a). Notably, the ICB fluorescence signal in blood circulation dissipated within 2 min post-injection, while the ICG signal persisted for up to 5 min. These results are consistent with their respective blood half-lives of 0.96 min for ICB and 4.64 min for ICG. After systemic blood circulation, the fluorescence signals of both dyes in the vasculature significantly decreased. Importantly, higher background signals remained detectable even 60 min post-injection with ICG, but not with ICB (yellow arrows).

This faster clearance of ICB was also evident in cholangiography imaging (Figure 3b). The ICB fluorescence signal moved from the bile duct to the intestine as early as 2 min post-injection, whereas ICG fluorescence in these areas was observed only after 10 min. Previous studies have shown that ICG often requires over 10–20 min to reach sufficient levels in the biliary tract for measurement following intravenous injection [55]. While ICG-assisted NIR cholangiography is a promising technique for enhancing the visibility of extra-biliary structures [56,57], the pharmacokinetics of ICG are less than ideal for real-time imaging in cases of acute biliary injury or intraoperative complications, such as unintended leaks, ductal injury, or accidental ligation [55]. Additionally, ICG tends to produce significant hepatic fluorescence, limiting the signal-to-background ratio (SBR). For optimal biliary imaging, NIR agents should, ideally, have fast and efficient biliary excretion to maximize SBR. As our results indicate that ICB offers sharper imaging of biliary structures than ICG, positioning ICB as a promising agent for immediate biliary imaging during procedures such as cholecystectomy (Figure 3b).

Immediate lymphatic mapping was also achieved using ICB. Following the hind footpad injection of ICB and ICG, the popliteal lymph node was visualized with the NIR imaging system, as shown in Figure 3c. The fluorescence signal from ICB increased, reaching maximum intensity at 1 h post-injection, and gradually diminished, becoming undetectable at 8 h (Figure 3d). In contrast, the fluorescence signal of ICG continued to increase until 2 h post-injection. Although ICB fluorescence intensity was slightly lower than ICG, ICB enabled rapid detection of the popliteal lymph node (~5 min) and exhibited faster clearance, with fluorescence almost gone by 8 h post-injection. This faster clearance of ICB from the lymph nodes aligns with pharmacokinetics and imaging results from angiography and cholangiography despite the different injection routes.

Altogether, these in vivo imaging analyses visually confirmed the pharmacokinetic properties of ICB, including rapid target detection and swift clearance from the body, attributable to its shorter blood half-life.

**Figure 4:**
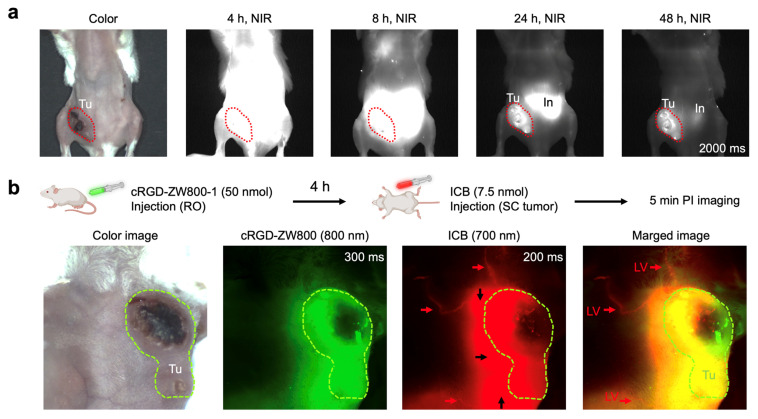
Tumor targeting of ICB in 4T1 breast tumor-bearing animal models. (**a**) Mice weighing 25 g were injected with 50 nmol of ICB, and their tumor signals were observed up to 48 h post-injection. (**b**) Simultaneous intraoperative dual-channel imaging of tumors and lymphatic vessels (LV; red arrows). A total of 7.5 nmol of ICB (red) was injected intratumorally (black arrows) 4 h post-injection of cRGD-ZW800-1 (green). Abbreviations used are In, intestine; LV, lymphatic vessel; Tu, tumor.

**Dual-Channel Imaging of Tumor and Lymphatic Vessels:** The successful application of ICB in visualizing lymph nodes, blood circulation, and biliary clearance in imaging experiments prompted us to explore more clinically relevant imaging, such as tumor imaging and tumor-associated lymphatic vessel visualization. A tumor model was established by orthotopically implanting 4T1 breast cancer cells into the right 4th mammary fat pad of BALB/c mice (Figure 4a). To assess the potential of ICB for tumor targeting, mice received a retro-orbital injection of ICB (50 nmol), and the tumor fluorescence signal was monitored over 48 h. High background signals, especially in the intestinal region, persisted until 24 h post-injection, with a distinct tumor signal emerging after 48 h. These results indicate that ICB could serve as an effective tumor imaging dye, offering a shorter wavelength alternative to ICG.

Next, we conducted a more complex dual-channel imaging experiment for simultaneous visualization of the tumor and its lymphatic vessels using a binary admixture of fluorescent probes with distinct emission wavelengths and targeting profiles. This dual-channel imaging setup paired ICB, a 700 nm fluorescent probe, for lymphatic vessel visualization with cRGD-ZW800-1, an 800 nm emitting tumor-targeting fluorophore [58]. To account for differing pharmacokinetics, the probes were administered sequentially. Mice were first injected retro-orbitally with the tumor-targeting cRGD-ZW800-1 (50 nmol), and 4 h later, ICB (7.5 nmol) was injected intratumorally. Imaging was performed in live mice using specific filters for each probe (700 nm for ICB and 800 nm for cRGD-ZW800-1). As shown in Figure 4b, images captured 5 min post-ICB administration revealed clear visualization of lymphatic vessels around the tumor tissue. This proof-of-concept study underscores the potential of ICB for rapid lymphatic vessel visualization around cancerous tissues stained with a cancer-targeting NIR probe cRGD-ZW800-1. These findings offer valuable insights for preclinical studies investigating changes in lymphatic drainage with tumor progression or treatment, and this approach could ultimately be integrated into clinical protocols for precise lymphatic dissection during cancer surgery.

## 3. Discussion

Our study builds upon the previous work of Smith and colleagues [52] by providing additional and compelling evidence for ICB as a potent NIR fluorophore with promising applications in image-guided surgery, presenting a viable alternative to ICG. ICB demonstrates a 100 nm shorter maxima absorption and fluorescence wavelength across all aqueous solvents examined, making it particularly suited for clinical imaging within the shorter 700 nm channel. While ICB’s aqueous solubility was slightly lower than that of ICG, its extinction coefficients were comparable to or higher in saline and buffer solutions (PBS and HBS). Additionally, ICB displayed a consistently higher quantum yield in all tested aqueous solutions, which is beneficial for achieving clear in vivo imaging with a high SBR. In fact, CCD cameras have 1.5- to 2-fold higher sensitivity at 700 nm compared to 800 nm, which improves ICB performance even further [40].

The biodistribution patterns of ICB were similar to ICG, while in contrast to ICG, an intriguing feature of ICB was its rapid clearance from the body, enabling the minimization of background signals, especially from the liver. This suggests that the potential advantage of ICB compared with ICG is that it can be utilized for cholecystectomy and primary and metastatic tumors in the liver due to its lower background. In addition, rapid imaging by ICB for angiography and cholangiography would also be beneficial during surgery. This rapid clearance of ICB might be due to its shorter polymethine bridge and lower molecular weight, a trend that is consistent with our previous report for the zwitterionic ZW700 and ZW800 series [46,59]. Generally, fluorophores with short polymethine, such as pentamethine ZW700-1, show faster distribution and elimination half-life values, in addition to a decreased area under the curve compared with heptamethine fluorophores. The rapid clearance of ICB may offer advantages in reducing adverse reactions. While ICG is generally regarded as safe and is widely used in clinical imaging, it can cause adverse effects, such as urticaria, itching, nausea, and vomiting [60]. Due to the structural similarity between ICB and ICG, combined with the faster clearance of ICB, it is possible that ICB could minimize these adverse reactions. However, further studies are needed to evaluate the safety profile of ICB before its clinical application. By employing fluorophores emitting at two separate wavelengths, we can significantly enhance tumor diagnostics, facilitate AI-based surgical training, and improve the safety of surgical interventions.

While rapid clearance might raise concerns about tumor selectivity due to reduced blood circulation time, we confirmed the tumor-targeting capability of ICB using an orthotopic breast cancer mouse model, where specific accumulation in tumor tissue was observed within 24 h post-injection. This is not surprising since several reports have demonstrated excellent tumor targetability of ICG [61,62,63,64]. However, since several fluorophores for 800 nm channels, including ICG, have been well-established in the detection of tumors [51,65,66,67], we highlighted that the usage of ICB as a 700 nm channel fluorophore is more beneficial for other purposes, such as visualizing blood vessels and lymphatic vessels during surgery for tumor resection, as well as lymphangiography. In this study, we designed two sets of NIR fluorophores and explored the use of two independent wavelengths of NIR fluorescence to provide simultaneous imaging of tumors by cRGD-ZW800-1 and nearby lymphatic vessels by ICB. Our results clearly demonstrated the significant potential of utilizing ICB for rapid visualization of lymphatic vessels around cancerous tissues that were simultaneously visualized using a tumor-targeting fluorophore (cRGD-ZW800-1). These findings suggest that novel dynamic contrast-enhanced imaging methods could be developed using the paired-fluorophore imaging paradigm.

## 4. Materials and Methods

### 4.1. Reagents and Materials

Indocyanine blue™ (ICB, aka NK-1841; CAS #64285-36-5, Hong Kong, China) was provided by Curadel Pharma, Inc. (Natick, MA, USA). Acetic acid, ethylene glycol, and bovine serum albumin (BSA) were purchased from Millipore Sigma (Burlington, MA, USA). ICG, phosphate-buffered saline (PBS), 4-(2-hydroxyethyl)-1-piperazineethanesulfonic acid (HEPES) buffer, fetal bovine serum (FBS), and saline were purchased from ThermoFisher Scientific (Waltham, MA, USA). The mouse breast cancer cell line 4T1 cells were purchased from ATCC (Manassas, VA, USA).

### 4.2. Physicochemical Property Measurements of ICB and ICG

The optical properties, including the absorbance and fluorescence spectra of the fluorophores, were measured using a UV-Vis-NIR spectrophotometer (USB-ISS-UV/VIS, Ocean Optics, Dunedin, FL, USA). The working solutions with a concentration of 5 µM in PBS, 10% FBS solution supplemented with 50 mM HEPES, pH of 7.4 (HBS, Boston, MA, USA), or dextrose 5% in water (D5W) were prepared from 10 mM stock of ICB and ICG in DMSO. About 2 mg of each fluorophore was dissolved in a minimal volume of water, PBS, HBS, and D5W to measure solubility. After 10 min of sonication and subsequent centrifugation, the saturated supernatant was collected, and its absorbance spectrum was measured and implemented in the corresponding standard curve of absorbance versus concentration. The physicochemical properties of fluorophores were calculated by using the MarvinSketch calculator plug-in (ChemAxon, Budapest, Hungary).

### 4.3. Quantum Yield Measurements

For fluorescence quantum yield (QY) measurements, oxazine 725 in ethylene glycol (QY = 19%) and ICG in PBS (QY = 2.7%) were used as calibration standards under conditions of matched absorbance at 660 nm [68] and 760 nm [53], respectively. The fluorescence spectrum of each dye was obtained with excitation at 660 or 760 nm, and the integrated area was used in the quantum yield calculation [53].

### 4.4. Serum Binding Assay

The serum binding assay was performed with slight modifications to the method we previously reported [59]. Working solutions with a concentration of 5 µM in PBS with 10% FBS/PBS were prepared from 10 mM of dye stock solutions in DMSO and added into the assigned sample chambers in a rapid equilibrium dialysis (RED) device along with PBS as the dialysis buffer. The device was covered and incubated at 37 °C on an orbital shaker at 20 rpm for 4 h. After incubation, the absorbance and fluorescence profiles of each fluorophore were measured using a plate reader (Bio Tek, Winooski, VT, USA) to calculate the concentrations of dyes in both chambers separately. The percentage of free and bound dye was calculated for each fluorophore.

### 4.5. NIR Fluorescence Imaging System

For dual-channel NIR imaging, 630 nm excitation light (700 nm NIR) at 1.0 mW/cm^2^ and 760 nm excitation light (800 nm NIR) at 1.0 mW/cm^2^ were used with white light (400–650 nm) at 5500 lux in the FLARE imaging system. Simultaneous color images (512 × 512 pixels) with the choice of either 700 or 800 nm fluorescence images were acquired using an AD-130GE camera (JAI, Yokohama, Japan) installed with a custom dual bandpass prism (channel #1: 710/50, bandpass filter, channel #2: 780 long pass filter). The imaging system was controlled by custom software at rates up to 15 Hz, except for the field of view, which was manually adjusted by a macro zoom lens (0–10×; Navitar Zoom 7000 with a SWIR coating; Rochester, NY, USA). In the color–NIR merged image, fluorescence images at 700 nm and 800 nm were pseudo-colored red and green, respectively. The imaging head was positioned at a distance of 9 inches from the surgical field, and all NIR fluorescence images have identical exposure times and normalizations.

### 4.6. In Vivo Biodistribution and Pharmacokinetics of ICB and ICG

The animals were housed in an AAALAC-certified facility and were studied under the supervision of MGH IACUC in accordance with the approved institutional protocol (2016N000136). Prior to the injection of treatments, six-week-old CD-1 mice (Charles River Laboratories, Wilmington, MA, USA) were anesthetized with isoflurane and oxygen, and blood was sampled in capillary tubes (Fisher Scientific, Pittsburgh, PA, USA) at time point 0 min by slightly cutting the end of the tail. ICB and ICG in saline were intravenously injected at the same dose level as the imaging experiments. Blood samples were obtained at 1, 3, 5, 10, 30, 60, 120, 180, and 240 min post-injection, and the fluorescence intensities of serum samples in capillary tubes were measured to calculate plasma half-life (*t*_1/2_) values (*n*  =  3 for each group). At 4 h post-injection, mice were sacrificed for image biodistribution and their organs (liver, lung, spleen, kidney, stomach, brain, intestine, and bladder) were resected. The fluorescence and background intensities of a region of interest over each tissue were quantified using customized imaging software and ImageJ v1.48 (National Institutes of Health, Bethesda, MD, USA). The signal-to-background ratio (SBR) was calculated as SBR = fluorescence/background. Results were presented as a bi-exponential decay curve using Prism software version 9.0 (GraphPad, San Diego, CA, USA).

### 4.7. Angiography

CD-1 mice were maintained under anesthesia with isoflurane and oxygen during the experiment. An arc-shaped incision was made in the abdominal skin. The connective tissue was separated to free the skin flap without injuring the artery and vein. Mice were intravenously injected with 100 µL of ICB or ICG (250 mM in saline) for angiography. NIR images of blood vessels on abdominal skin were obtained until 1 h post-injection.

### 4.8. Cholangiography

CD-1 mice were maintained under anesthesia with isoflurane and oxygen during the experiment. For open-surgery experiments, a standard midline laparotomy was performed to expose the gallbladder and bile duct. Mice were intravenously injected with 100 µL of ICB or ICG (250 mM in saline) for cholangiography. NIR images of the gallbladder and bile duct were obtained until 1 h post-injection.

### 4.9. Lymph Node Mapping

CD-1 mice were maintained under anesthesia with isoflurane and oxygen during the experiment. The mice’s fur on the region of interest was shaved prior to imaging using a clipper and removed completely using a depilatory cream. Either 10 µL of ICB or ICG (250 mM in saline) were injected from the hind paw. NIR images of the popliteal lymph node were obtained until 24 h post-injection.

### 4.10. Tumor and Lymphatic Vessel Dual-Channel Imaging

Mice were maintained under anesthesia with isoflurane and oxygen during the experiment. To establish the orthotopic breast cancer mouse models, eight-week-old female Balb/c mice (Charles River Laboratories, Wilmington, MA, USA) were orthotopically injected with 5 × 10^5^ 4T1 cells suspended in PBS (50 µL). Before the experiment, tumors were allowed to reach a size of approximately 600 mm^3^. Once tumor volumes reached around 600 mm^3^, mice were intravenously injected with 100 µL of ICB (1000 mM) for tumor targeting and cRGD-ZW800-1 (500 mM) for dual-channel imaging, respectively. At 24 h following the cRGD-ZW800-1 injection, 10 µL of ICB (250 mM) was injected subcutaneously into the tumor region for lymphatic vessel imaging.

### 4.11. Statistical Analysis

The fluorescence and background intensities of a region of interest over each tissue were quantified using customized imaging software and ImageJ v1.48 (National Institutes of Health, Bethesda, MD, USA). The signal-to-background ratio (SBR) was calculated as SBR = fluorescence/background, where the background is the fluorescence intensity of the muscle. Data are reported as a mean ± s.e.m., with a minimum of three biological replicates. A Student’s *t*-test statistical analysis was performed to evaluate the significance of the experimental data. We also used a one-way ANOVA followed by Tukey’s multiple comparisons test to compare the results among more than two groups. A *p*-value of less than 0.05 was considered significant. The data was indicated with * *p* < 0.05, ** *p* < 0.01, and *** *p* < 0.001.

## 5. Conclusions

Our study demonstrates that ICB is a powerful NIR fluorophore for applications in angiography, cholangiography, lymph node and lymphatic vessel mapping, and tumor detection, optimized for shorter wavelength (700 nm) channels. Remarkably, the modification of a single methine unit in the polymethine backbone alters its optical properties, enhancing rapid circulation and biliary excretion, which results in immediate imaging with lower background signals. These characteristics enable ICB to be effectively utilized in dual-channel imaging alongside ICG, which is already FDA-approved and widely used for various clinical imaging applications. Our findings pave the way for advancing dynamic contrast-enhanced imaging, leveraging paired or multimodal agent imaging paradigms for improved diagnostic and surgical outcomes.

## Figures and Tables

**Table 1:** Physicochemical properties of ICB and ICG in DW, saline, PBS, HBS, and D5W ^[a]^.

Dye	Solvent	Solubility (mg/mL)	λ_Abs_ (nm)	λ_Em_ (nm)	SS (nm)	*ε* (M^−1^ cm^−1^)	QY (*ϕ*, %)
ICB	DW	3.58	678	708	30	211,000	5.3
	Saline	0.33	676	705	29	186,000	4.7
	PBS	0.28	677	706	29	183,000	4.3
	HBS	0.63	696	728	32	210,000	17.0
	D5W	3.11	678	706	28	185,000	2.2
ICG	DW	3.25	778	815	37	225,000	2.8
	Saline	0.51	778	810	32	140,000	2.3
	PBS	0.45	779	811	32	139,000	2.6
	HBS	2.71	800	830	30	174,000	16.0
	D5W	3.21	779	814	35	200,000	1.0

^[a]^ DW, distilled water; PBS, phosphate-buffered saline with a pH of 7.4; HBS, 10% fetal bovine serum supplemented with 50 mM HEPES with a pH of 7.4; D5W, 5% dextrose supplemented in water. λ, wavelength; SS, Stokes shift; *ε*, extinction coefficient; QY, quantum yield.

## Data Availability

All data are available in the main text.
